# Electrically Tunable Solution-Processed Transparent Conductive Thin Films Based on Colloidally Dispersed ITO@Ag Composite Ink

**DOI:** 10.3390/nano12122060

**Published:** 2022-06-15

**Authors:** Yoo Lim Cha, Jeong-Hye Jo, Dong-Joo Kim, Sun Hee Kim

**Affiliations:** 1 Materials Research and Education Center, Department of Mechanical Engineering, Auburn University, Auburn, AL 36849, USA; yzc0133@auburn.edu; 2 Department of Materials Science and Engineering, Gachon University, Seongnam 13120, Korea; mj06229@gachon.ac.kr; 3 Department of Fashion Industry, Incheon National University, Incheon 22012, Korea

**Keywords:** transparent conductive oxides, silver, Sn-doped In_2_O_3_, colloid, spin coating

## Abstract

Silver (Ag) introduced colloidal Sn-doped In_2_O_3_ (ITO) ink for transparent conductive electrodes (TCEs) was prepared to overcome the limitation of colloidally prepared thin film; low density thin film, high resistance. ITO@Ag colloid ink was made by controlling the weight ratio of ITO and Ag nanoparticles through ball-milling and fabricated using spin coating. These films were dried at 220 °C and heat-treated at 450–750 °C in an air atmosphere to pyrolyze the organic ligand attached to the nanoparticles. All thin films showed high crystallinity. As the thermal treatment temperature increased, films showed a cracked surface, but as the weight percentage of silver increased, a flattened and smooth surface appeared, caused by the metallic silver filling the gap between the nano-particles. This worked as a bridge to allow electrical conduction, which decreases the resistivity over an order of magnitude, from 309 to 0.396, and 0.107 Ω·cm for the ITO-220 °C, ITO-750 °C, and ITO@Ag (7.5 wt.%)-750 °C, respectively. These films also exhibited >90% optical transparency. Lowered resistivity is caused due to the inclusion of silver, providing a sufficient number of charge carriers. Furthermore, the work function difference between ITO and silver builds an ohmic junction, allowing fluent electrical flow without any barrier.

## 1. Introduction

Transparent conducting oxides (TCOs) represent an essential component in numerous electronic devices, including organic optoelectronic devices, such as photovoltaic cells and light-emitting diodes, sensors, and transistors [1,2,3,4,5,6,7,8,9]. The commonly used transparent conducting oxide is indium-tin-oxide (ITO) due to its superior transparency in the visible range (transparency, T > 85%) and high electrical conductivity (resistivity, $\rho =\left(1\sim 2\right)\times 10^{-4}\;\Omega \cdot \mathrm{cm}$). Although ITO is the most commercially used transparent conducting material as an optoelectronic electrode, it still has some drawbacks to overcome.

Fundamentally, the properties of the ITO electrode are strongly affected by its fabrication process. Currently, the physical vapor deposition (PVD) process, especially the radio frequency (RF) or direct current (DC) sputtering method, is the traditional way to attain ITO electrodes with high transparency and conductivity on an industrial scale. This is because PVD techniques can grow the epitaxial and high-density TCO coatings/films with outstanding performances in transmittance and conductivity. Unfortunately, these methods demand a high vacuumed system, which causes the equipment and parts to be high-priced. Consequently, the TCOs deposition process becomes expensive, limiting its compatibility with mass production and lowering its accessibility to numerous types of businesses. It also makes it challenging to keep up with the industrial trends of making large-scale, low-cost devices. Furthermore, much of the deposition material is wasted since it is deposited on the chamber walls [10].

As an alternative method, solution-based wet chemical processes, e.g., dip coating [11,12], spin coating [13,14], doctor blading [15,16], bar coating [17,18], and slot-die coating [19,20], to fabricate thin film have acquired great research attention in recent years due to their fascinating features. They need only simple components for constructing equipment since the deposition process is done at atmospheric pressure, making the price of deposited electrodes less expensive. Moreover, according to the fabrication techniques, it is feasible to coat larger substrates and substrates with different geometries, which allows fulfilling the industrial requirements. The solution-based thin film deposition methods can be divided into three types based on their precursor ink: sol-gels, inorganic nanoclusters, and colloidal nanoparticles. Especially the colloidal ink, which is also called nano-particle suspension ink, has several particular merits compared to other inks, in which the sizes, morphologies, compositions, and dispersibility of TCO nanoparticles can be easily manipulated. Moreover, its methods for producing ink, i.e., ball-milling, are relatively simple processes compared to others. Therefore, it is not extremely sensitive to experimental variables, making it suitable for mass production with high reproducibility. In addition, the films deposited through this ink are entirely crystalline since it includes a relatively low carbon and salt pollution source. Furthermore, its surface can be made suitable for various polar and nonpolar solvent systems.

However, long-chain surfactants called coupling ligands are necessary to maintain the colloidal state, which the nanoparticles keep suspended and prevent agglomeration of the nanoparticles. In addition, it needs a thermal decomposition process to eliminate organic ligands attached to the TCOs surface, which cause higher porosity and higher density of grain boundary of the films than the others. This can cause a lowered electron mobility and higher resistivity since the boundary acts as a scattering matter to reduce the mean free path of the electron and presents a higher energy barrier for electronic conduction [21].

Several researchers are trying to overcome these drawbacks through several methods, such as synthesizing monodisperse TCO particles for self-assemblies [22,23] and controlling the morphologies or sizes of the particles [24,25]. However, to the best of our knowledge, the introduction of silver metals to form a composite with ITO for colloidally dispersed ink has not been studied. Herein, we investigate the influence of the compositional effect of the ITO and silver (Ag) on the optoelectronic properties by producing colloidal ink using a series of weight-ratio controlled Sn-doped In_2_O_3_ (ITO) and Ag nanocrystals. This research suggests a method to overcome the fundamental limitations of colloidal ink; low density, low electrical conductivity, that cannot be overcome through previous research methods; morphology and size control, by forming a colloidal ink consisting of ITO and metal (Ag), which can provide excessive carriers and has high electrical conductivity.

## 2. Materials and Methods

### 2.1. Materials

The commercially purchasable Indium tin oxide, ITO, nanopowder (90:10 wt.%, 99.5%, 17–28 nm, Alfa Aesar, Haverhill, MA, USA), and silver powder (Spherical, 0.5–1 um, 99.9%, Alfa Aesar, USA) were used as conductive materials. Ethanol (Anhydrous, 99.9%, Samchun, Seoul, Korea), DMF (N,N-Dimethylformamide, (CH_2_)_2_NCH, 99.9%, Sigma-Aldrich, Burlington, MA, USA), and IPC (2-isopropoxyethanol, C_3_H_8_O, 99%, Sigma-Aldrich, St. Louis, MO, USA) were used as a dispersing medium. Moreover, to form a solvation force between colloidal nano-particles, titanate coupling agent, KR-44 (Isopropyl tri(N-ethylenediamino)ethyl titanate, Hansol Fine, Seoul, Korea, Kenrich Petrochemicals, Bayonne, NJ, USA) was used as a dispersant. ZrO_2_ ball with 0.3 mm diameter (Cenotec, Cheongju, Korea) was used to make colloidally dispersed ink by dispersing ITO or ITO and silver nanoparticles in the dispersing medium during the high-speed ball milling process.

### 2.2. Production of ITO@Ag Colloidal Sol

The colloidal suspension of ITO@Ag consists of 5 wt.% of conductive powder and 95 wt.% of organic solvent, which is the weight ratio of ethanol:DMF:IPC = 87.65:3.53:8.82. The coupling agent was used as a ligand surrounding the conductive nanoparticles to make them repel each other, and its amount was set as 44.7 wt.% of conductive materials. To investigate the effect of silver, a series of conductive materials with different weight ratios were set as 100:0, 97.5:2.5, 95:5, 92.5:7.5 (ITO:Ag). The higher weight percentage of silver was not considered in this research since as the silver content increases, it was considered that the cost merit of the solution-processable electrode disappears. After weighing these precursors, all of these were mixed thoroughly with a magnetic stirrer. Mixed precursors were ball milled for 7 h with 0.3 mm zirconia beads with planetary milling (Pulverisette 5/4, Fritsch, Idar-Oberstein, Germany).

### 2.3. ITO@Ag Transparent Electrode Fabrication

Prior to fabricating the ITO@Ag films, the glass (Soda-lime, 25 mm × 25 mm × 0.5 mm) and quartz substrates (fused silica, silicon dioxide (SiO_2_)), 15 mm × 15 mm × 0.5 mm) were wet cleaned/sonicated with commonly used solvents; ethanol, acetone, and deionized water in sequence for 5 min each and blow-dried with nitrogen. Then pre-cleaned substrates were dry-cleaned with an O_2_ plasma ion generator (CUTE-1MPR, Femto Science, Hwaseong, Korea) for 3 min with a 100 W, working pressure of 2.0 torr and a gas flow rate of 20 sccm. Directly after the plasma treatment, the prepared substrates were spin-coated with the ITO@Ag colloidal solutions at 1500 rpm for 90 sec. Then, deposited films were dried on a hot plate at 220 °C for 1 min to remove dispersing solvent. Subsequently, the films were thermally treated in a thermal process furnace (JB-14P, J-one, Seongnam, Korea) at 450, 600, and 750 °C for 3 h with a 3 °C/min heating rate in atmospheric conditions. In order to prevent side reactions at higher temperatures just in case, the temperature variable has been set up to 750 °C. Since the glass substrate has a glass transition from 564 °C, quartz substrates were used for ITO@Ag electrode samples heat-treated at 600 °C and 750 °C to avoid side reactions.

### 2.4. Characterization

The crystalline phase of the ITO@Ag thin films was determined by using X-ray diffraction (XRD, Rigaku, SmartLab, Japan) with nickel-filtered Cu Kα radiation (λ = 1.5418 Å), operating on a *θ/2θ* configuration at 40 kW and 40 mA. The topology of the sample was obtained using scanning electron microscopy (SEM, S-4700, Hitachi, Tokyo, Japan) operating at 15 kV accelerating voltage. The transmittance of the thin films was measured by a UV-Vis Spectrophotometer (V-750, Jasco, Tokyo, Japan). The sheet resistance of the ITO@Ag thin films was obtained by four-point probe measurements and the van der Pauw method over the 18 mm × 18 mm glass substrate at room temperature. The Hall effect was measured by the van der Pauw method. The thermal deposition temperature of the coupling agent was analyzed using thermogravimetric analysis and differential Scanning Calorimetry measurements (TGA/DSC, SDT Q600, Ta Instruments, New Castle, DE, USA). The high viscous coupling agent was placed in the alumina crucible and heated with 10 °C/min increments under a 100 mL/min nitrogen flow from room temperature to 600 °C. An empty alumina crucible was used as a reference. The surface chemical state analyses of ITO and ITO@Ag thin films were conducted through a K-Alpha X-ray photoelectron spectroscopy (XPS) system (Thermo Fisher Scientific, Waltham, MA, USA) equipped with a 180° double-focusing hemispherical analyzer and monochromated Al Kα (1486.6 eV) radiation. The position of C 1s (284.8 eV) is used as the calibration standard to determine the accurate electron binding energies.

## 3. Results and Discussion

### 3.1. Structural, Morphological, and Compositional Properties of ITO@Ag Thin Film

**Figure 1:**
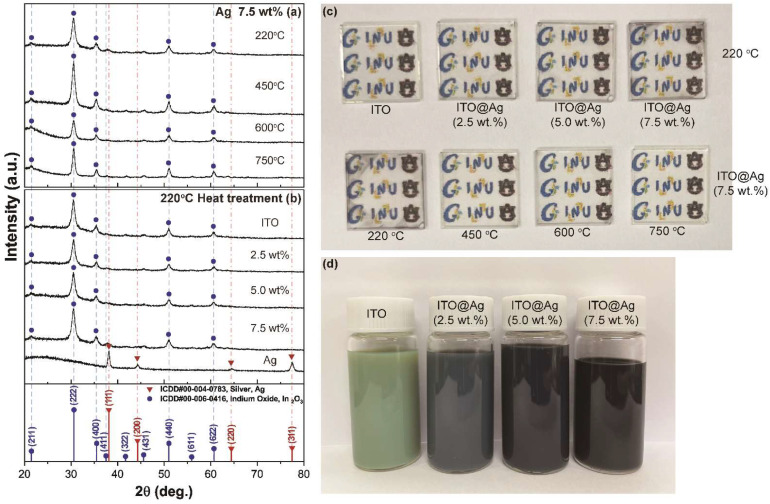
XRD patterns of ITO@Ag (7.5 wt.%) thin films according to (**a**) thermal-treatment temperature, (**b**) thin films heat-treated at 220 °C according to the weight percentage of silver, and ICDD PDF data of Ag (00-004-0783) and In_2_O_3_ (00-006-0416) (**c**) photograph of the deposited electrodes according to the weight percentage of silver and thermal-treatment temperature, (**d**) photograph of colloidal ITO and ITO@Ag ink.

The phase configuration of the ITO@Ag thin films was evaluated using XRD. Figure 1a shows the X-ray diffraction patterns of ITO@Ag thin films, including 7.5 wt.% of Ag, according to the heat treatment temperature changes. Diffraction peaks at 2θ = 21.5°, 30.6°, 35.5°, 51.0°, and 60.7° in XRD patterns are all matched to cubic bixbyite crystalline structure of In_2_O_3_ coordinated with ICDD card number 00-006-0416 (a=b=c=10.118 Å, space group Ia3 (206)). Furthermore, a broad hump between 20° ≤ 2θ ≤ 35° can be ascribed to the amorphous characteristics of soda-lime glass and fused silica substrates. Since different substrates were used according to the heat-treatment temperature, these humps also show different shapes and intensities.

Meanwhile, the corresponding mean crystallite sizes were calculated using the Scherrer equation

(1) $$D=\frac{K\lambda }{\beta \cos\theta }$$

where *D* is the mean grain size, β is the full width at half-maximum peak broadening in radian, K is a constant, and the value is 0.89 here, λ is the wavelength of X-rays (1.5418 Å), and θ is the angle of diffraction. The mean crystallite sizes calculated through the main peak reflected by the (222) plane were 9.51 nm, 10.1 nm, 11.0 nm, and 13.6 nm from 220 °C, 450 °C, 600 °C, and 750 °C, respectively, increasing along with the heat treatment temperature rises. This results from the high temperature providing additional thermal energy needed to overcome the energy barrier required for atomic diffusion, which results in aggregations of smaller particles with bigger particles [26]. However, diffraction peaks from silver or other characteristic peaks, such as silver compounds (ex. Ag_2_O), were not revealed in all temperature ranges. Moreover, no other peak shift of ITO peaks was observed, which implies that the amount of introduced silver is small enough to be the intensity of the peak extremely weak, and its particles are tiny and highly distributed or dispersed in the ITO sol.

The photograph of thin films prepared according to the weight percentage of silver and thermal treatment temperatures and prepared colloidal inks are in Figure 1c,d, respectively. The colloidal ink formed by high-speed ball milling (Figure 1d) does not form any aggregation or sedimentations over time, confirming that stable steric repulsion between conductive nanoparticles is formed. It shows a great significance in that it is possible to make stable colloidal ink by dispersing nanoparticles in a powder state that has already been finished synthesizing by utilizing a relatively long organic ligand, which is suitable for mass production and reproducibility. However, a long organic ligand requires a relatively high-temperature pyrolysis process to remove it from the thin film, which can prohibit the chance of being applied in flexible electronics. Therefore, it is considered that further research on attaching a shorter length of the organic ligand with a lower thermal decomposition temperature is necessary for the application of this method to flexible substrates.

All thin films fabricated with these colloidal inks show high transmittance in the visible light region (Figure 1c). For the thin film dried at 220 °C, the thin film turns slightly black as the silver content increases. The same trend appears for the colloidal inks (Figure 1d). However, the thin films using ITO@Ag (7.5 wt.%) become transparent while the black color disappears as the temperature increases. Explanations for these will be described in detail through the XPS analysis results below.

The ITO@Ag thin films after the drying process at 220 °C according to the weight percent of silver were also analyzed using XRD as presented in Figure 1b. All the patterns show high crystalline ITO peaks. Moreover, as in Figure 1a, no silver diffraction peak was found in all silver weight percentages. For comparison, silver nanopowder used as a precursor for the colloidal ink was also analyzed, which shows the diffraction peaks positioned at 2θ = 38.12°, 44.28°, 64.43°, and77.47° corresponding to the (111), (200), (220), and (311) reflections of face-centered cubic (fcc) silver (ICDD card no. 00-004-0783, a=b=c=4.086 Å, space group $Fm\bar{3}m$ (225)). This confirms that no silver peaks were detected from ITO@Ag thin films because of the small amount and even distribution.

**Figure 2:**
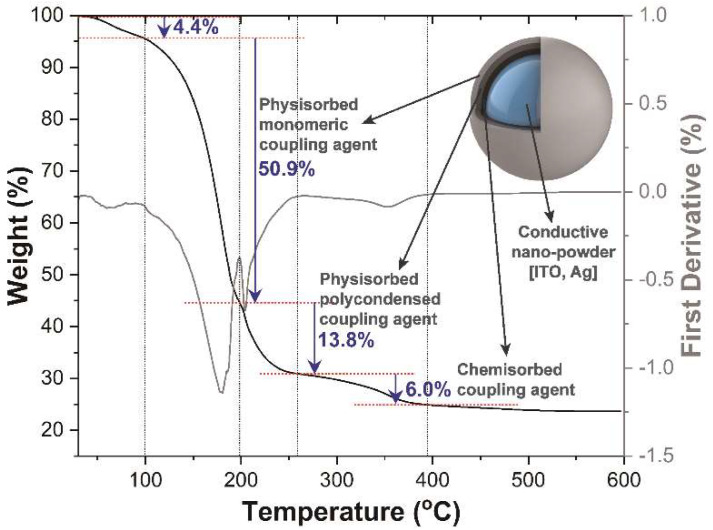
Thermal gravimetric analysis (TGA) and its first derivative curve for KR−44 coupling agent. Inset: schematic diagram of layered coupling agent distribution covering the conductive particle.

Since the coupling agent can prohibit the electrical connections between the conductive nanoparticles, the thermal decomposition behavior of the coupling agent was identified through TGA/DSC analysis as shown in Figure 2. In the result, the weight decreases occur in four steps: 30–99.4 °C, 99.4–198.3 °C, 198.4–258.6 °C, and 258.6–394.4 °C with 4.4%, 50.9%, 13.8%, and 6.0% of mass decreases respectively. The first mass loss is associated with the loss of physically adsorbed water from the surface [27]. The next three-step weight loss is shown as the same trend as the other literature [28], attributed to the thermal decomposition of physisorbed monomeric, physisorbed, polycondensed, and chemisorbed coupling agents having a configuration as shown in the schematic diagram from Figure 2 (inset). Through this analysis, it can be seen that the high-temperature pyrolysis process of 600 °C or 750 °C is required for improving electrical conduction by removing the dispersants covering the conductive ITO and silver nanoparticles.

**Figure 3:**
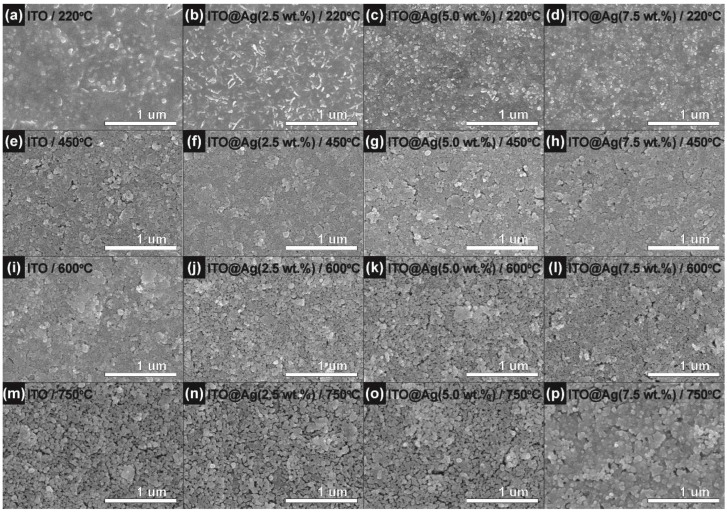
Scanning electron microscopy (SEM) images of the ITO and ITO@Ag thin films heat-treated at (**a**–**d**) 220, (**e**–**h**) 450, (**i**–**l**) 600, and (**m**–**p**) 750 °C according to the weight percentage of silver.

The topological changes of deposited films according to the thermal treatment temperature and weight percentage of silver were characterized through scanning electron microscopy (SEM). Figure 3a–d shows the surface structure of ITO (a), ITO@Ag (b–d) thin films dried at 220 °C in atmospheric conditions. These thin films show a smooth surface with minor bumps. These bumps increase with the weight percentage of silver, which is caused due to different grafting efficiency of coupling agents upon the surface of ITO and silver [29]. Despite this, the particles constituting the thin film do not cluster together but are uniformly dispersed so that the aggregation tendency of inorganic NPs is very small, and stable steric repulsion between the particles is formed. These smooth surfaces come from the boiling point of the dispersant used. In the drying process at 220 °C, all dispersants, DMF, IPC, and EtOH, can be evaporated due to their boiling points at 178.3, 143, and 78.37 °C, respectively. However, according to the TGA analysis (Figure 2), 35.2% of the mass from the coupling agent can still be left in the thin film at 220 °C. The remaining coupling agent can fill the gaps between the conductive nanoparticles and connects each other, so it shows the flat and even surfaces. When the ITO and ITO@Ag thin films were heat-treated at 450 °C (Figure 3e–h) and 600 °C (Figure 3i–l), the smoothness of the surface is getting disappeared because most of the coupling agent was calcined (about 24% left) and rough surfaces are getting revealed. Not only does the gap between secondary particles increase, but the size of the primary particles also increases in accordance with the crystallite size calculated through the XRD results. However, in the thin films heat-treated at 750 °C (Figure 3m–p), 7.5 wt.% of silver containing ITO@Ag thin film shows the smoothest surface and the densest contact between conductive particles in contrast to the thin films dried at 220 °C.

**Figure 4:**
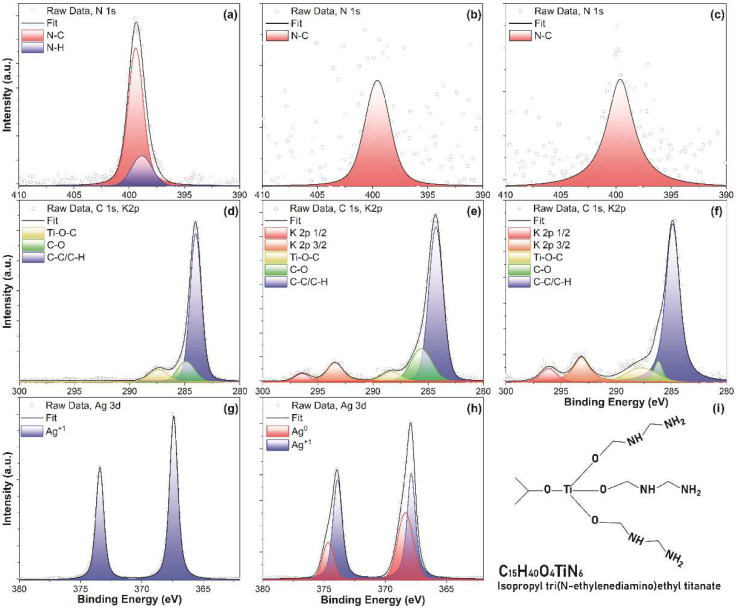
High-resolution X-ray photoelectron spectroscopy (XPS) spectra of N 1s for ITO@Ag(7.5 wt.%) thin film heat-treated at (**a**) 220 °C, (**b**) 750 °C, and (**c**) ITO thin film heat-treated at 750 °C, C 1s for ITO@Ag (7.5 wt.%) thin film heat-treated at (**d**) 220 °C, (**e**) 750 °C, and (**f**) ITO thin film heat-treated at 750 °C, Ag 3d for ITO@Ag (7.5 wt.%) thin film heat-treated at (**g**) 220 °C, (**h**) 750 °C, and (**i**) chemical structure of coupling agent.

For a more detailed explanation of this phenomenon, the chemical bonding configuration of ITO and ITO@Ag (7.5 wt.%) thin films were measured through the XPS (Figure 4). The N 1s peak of the (Figure 4a) ITO@Ag thin films containing 7.5 wt.% silver dried at 220 °C can be fitted with two peaks at 398.8 ± 0.1 and 399.4 ± 0.1 eV, corresponding to N-C and N-H bonding, respectively [30,31]. The N-H bond disappears through the thermal treatment of 750 °C (Figure 4b), and only the N-C bond remains. Moreover, the atomic percentage of nitrogen decreases by 92%, from 3.23 at.% to 0.25 at.% as the thermal treatment temperature increases from 220 °C to 750 °C, which matches well with the TGA result. This tendency can also be observed in ITO thin film heat-treated at 750 °C (Figure 4c) since the same coupling agent was used and it pyrolyzed and lost its weight. In addition, the ITO@Ag (7.5 wt.%) sample heat-treated at 750 °C (Figure 4b) has a lower intensity of the N 1s peak than the ITO heat-treated at 750 °C (Figure 4c). The reason is considered to be that Ag acts as a catalyst that promotes the thermal decomposition of the chemical bond of N [32]. The high-resolution spectrum of C 1s in ITO@Ag (7.5 wt.%) thin films dried at 220 °C (Figure 4d) could be deconvoluted into three peaks, which centered at 287.9 ± 0.1, 286.1 ± 0.1, and 284.8 ± 0.1 eV, which accordance with the Ti-O-C, C-O, and C-C/C-H, respectively [31]. All these structural features can be found in the chemical structure of the coupling agent (Figure 4i). In the XPS spectrum of the thin film containing 7.5 wt.% of silver (Figure 4d) and ITO thin film (Figure 4e) heat-treated at 750 °C, the intensities of K 2p 1/2 and K 2p 3/2 peaks at 296.1 ± 0.1, and 293.1 ± 0.1 eV rise since the impurities of the quartz substrate. Moreover, the atomic percent of carbon diminishes by 72% from 45.32 at.% to 12.74 at.% as the heat-treatment temperature rises from 220 °C to 750 °C, which is the result of the thermal decomposition process of the coupling agent. In the case of the silver, only the Ag^1+^ in Ag_2_O energy peaks appear from the 220 °C of pyrolysis (Figure 4g), which centered at 368.1± 0.1 and 374.2 ± 0.1 eV [33,34]. This is because the silver particles are oxidized during the high-energy dispersion process for colloidally dispersed ink. Whereas the peaks of Ag metal appear from the heat treatment at 750 °C on 368.6 ± 0.1, 374.6 ± 0.1 eV [35], which implies the Ag_2_O is thermally decomposed as the chemical reaction below [36].

$$2{\mathrm{Ag}}_{2}O+C\;\to 4\mathrm{Ag}+{\mathrm{CO}}_{2}$$

Therefore, the 7.5 wt.% of silver contained ITO@Ag thin film (Figure 3p) showed smoother topology and superior contacts between the conductive ITO and Ag nanoparticle even though most of the coupling agent is decomposed and calcined away at 750 °C. This can be ascribed to the reduction reaction from silver oxides to silver in high temperatures, which allows the silver to permeate and fill the gaps between the conductive particles.

### 3.2. Optical Properties of ITO@Ag Thin Film

**Figure 5:**
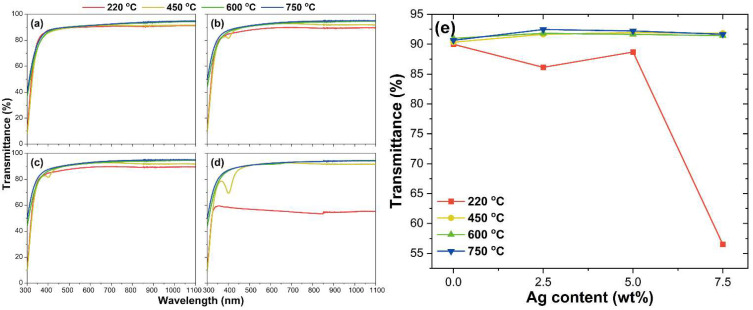
Optical transmittance spectra of (**a**) ITO, (**b**) ITO@Ag (2.5 wt.%), (**c**) ITO@Ag (5.0 wt.%), (**d**) ITO@Ag (7.5 wt.%) thin films, and (**e**) optical transmittance of thin films at 550 nm.

The optical transmittance of the ITO, ITO@Ag thin films was evaluated with UV-Vis spectroscopy, and its results are shown in Figure 5. The measurement was conducted according to the weight percentage of the silver and thermal treatment temperature in a wavelength range from 300 to 1100 nm. ITO thin film (Figure 5a) shows stably excellent transmittance, which is about 90% of transmittance at 550 nm in all temperature conditions. However, as the content of the silver increases, the transmittance tends to decrease when the thin films are post-heat treated in a low temperature (220 °C). This is because Ag_2_O present in the middle of the ITO particles is opaque in the visible light region even though it is evenly distributed. In addition, as the heat treatment temperature rises to 450 °C, the absorption peaks near 400 nm occur due to the Ag produced from the reduction of Ag_2_O [37,38]. These absorption peaks increase their intensity as the silver content increases. However, it disappears as the heat treatment temperature increases to 600 °C or 750 °C and shows excellent transmittance, similar to ITO, which shows a high transmittance value above 90% at 550 nm. This is because, as seen in SEM analysis (Figure 3p), ITO and silver agglomerate and form ITO@Ag secondary particles as the heat treatment temperature rises to form a smooth-surfaced thin film. Moreover, because this proceeded with the silver filling the gaps between the ITO particles, the thinly distributed silver does not affect the transmittance of the ITO [39]. The trend of the transmittance change of the thin films at 550 nm is summarized in Figure 5e.

The optical band gap of ITO and ITO@Ag thin films was calculated using the Tauc equation as written below [40]:

(2) $${\left(\alpha h\nu \right)}^{\frac{1}{n}}=A\left(h\nu -E_{g}\right)$$

where α is the absorption coefficient, h is the Plank’s constant, ν is the photon’s frequency, *A* is an energy-independent constant, *E_g_* is the optical band gap, and *n* is the index that denotes the nature of the electron transition process. It can be assigned to 1/2 for direct allowed band gap, 2 for indirect allowed band gap, 3/2 for direct forbidden transition, and 3 for indirect forbidden transition. Typically, the allowed transitions dominate the basic absorption processes [41], and for metals, the indirect interband transition may be disregarded since, in general, it is weaker than the direct interband transition by 2–3 orders of magnitude. So, only *n* = 1/2 was considered in this analysis. The absorption coefficient (α) is calculated using the equation [40].

(3) $$\alpha =\left(\frac{2.303}{d}\right)\log\frac{1}{T}$$

where *d* is the thickness of the thin film, 2.303 is the constant from Beer–Lambert law, and *T* is the transmittance measured with UV-Vis spectroscopy.

**Figure 6:**
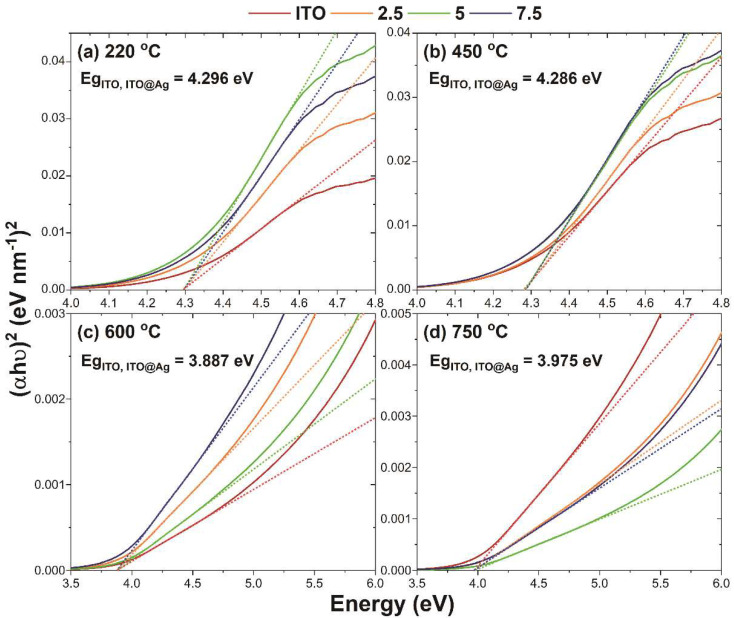
Tauc plot from absorption spectra of ITO and ITO@Ag thin films according to the thermal−treatment temperature (**a**) 220 °C, (**b**) 450 °C, (**c**) 600 °C, and (**d**) 750 °C. The straight dotted line extrapolation determines the bandgap.

The relationship between ${\left(\alpha h\nu \right)}^{2}$ against the photon energy (hν) of ITO, ITO@Ag thin films, according to the heat-treatment temperature rises, is presented in Figure 6. The optical band gap was calculated by fitting the straight lines to the linear region of the Tauc plots and extrapolating this region to find the *x*-axis intersection points, which are taken as the *E_g_* of the material. The calculated *E_g_* of ITO thin films coincides well with the literature value, ranging from 3.5 to 4.3 eV [42]. Moreover, all the fabricated thin films heat-treated showed the same *E_g_* value between the ITO and ITO@Ag thin film. This is because the silver itself does not contribute to the huge tune of the band gap since its amount is small enough. Moreover, as observed from XRD and SEM, when the thermal treatment temperature increases, the silver is distributed evenly and uniformly among the ITO particles, which means that the thinly formed silver layer between the ITO particles does not affect the optical properties of ITO. From 220 °C to 600 °C, the *E_g_* is continuously diminishing up to 3.88 eV. This is due to the ITO nanoparticles becoming aggregates and growths through the thermal treatment, which results in electric charges in the particle that can spread out of the nanoparticle and ends up showing decreased *E_g_* [43]. Since the calculated crystallite size and the primary particle size do not exceed 50 nm according to the Scherrer equation and SEM analysis, the particle size can affect the shift of *E_g_* [44]. However, as the heat-treatment temperature increases to 750 °C, it increases to 3.97 eV. This can be ascribed to increased carrier concentration due to the Moss-Burstein shift [45,46,47]. A detailed explanation of changes in carrier concentration will be discussed in the following section.

### 3.3. Electrical Properties of ITO@Ag Thin Film

**Figure 7:**
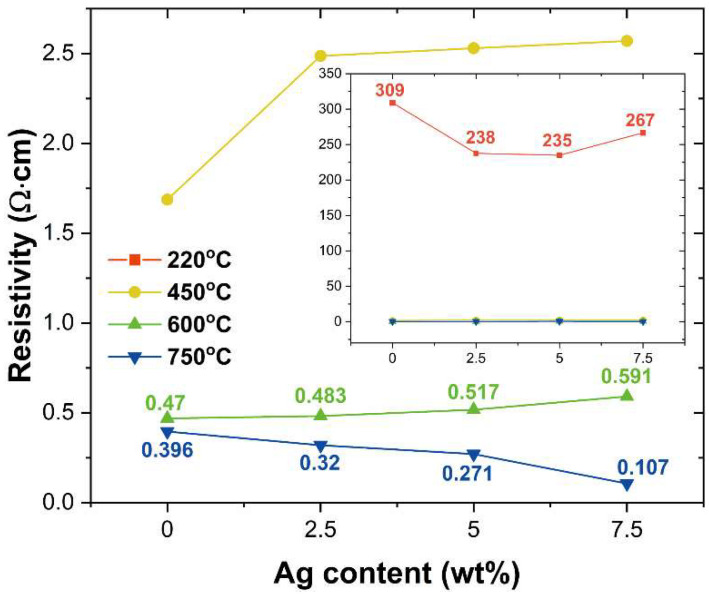
Magnified region of resistivity. Inset: overall resistivity of ITO and ITO@Ag thin films.

The electrical properties of the ITO and ITO@Ag thin films were evaluated through the 4-point probe and hall measurement. The measured resistivity (ρ) according to the weight percentage of silver and thermal treatment temperature is shown in Figure 7. The resistivity of the ITO thin films decreased from 309 to 0.396 Ω·cm as the thermal treatment temperature increased from 220 °C to 750 °C. For the case of the ITO@Ag (7.5 wt.%) thin films, the resistivity decreased from 267 to 0.107 Ω·cm. The decrease in resistivity is mainly due to the thermal decomposition of the coupling agent, as revealed in TGA analysis. Moreover, the resistivity of thin films tends to increase as the weight percent of silver increases from the thermal treatment temperature of 220 °C to 600 °C. This means that the inclusion of silver appears to have a negative effect on the electrical conductivity of the thin films at a low heat treatment temperature, which is 220, 450, and 600 °C here. The reason for this is that the Ag is dispersed in ITO as an oxide form, Ag_2_O, in accordance with the XPS results. Since Ag_2_O is the p-type semiconductor, a p-n junction would be constructed between the ITO and Ag_2_O. If considering the particles uniformly distributed in the n-p-n or p-n-p form, a reverse bias will be applied, which results in essentially no current flow unless a certain voltage, the so-called threshold voltage, is reached [48]. However, the difference between the resistivity of ITO and the one of ITO@Ag (7.5 wt.%) gets smaller as the temperature rises, which means the reduction of Ag_2_O to Ag shows a positive effect on the conductivity of the thin film. Since the amount of Ag metal produced during the reduction process would increase by increasing the temperature, the beneficial effect of Ag on conductivity will getting increase as the pyrolysis temperature rises and the weight percentage of silver increases, even though the pyrolysis process times are the same in all thermal treatments. The point where the silver content starts to have a positive effect is 750 °C, which can be explained by the XPS results (Figure 4g,h). According to XPS data, Ag_2_O still remains even after heat treatment at 750 °C, which means more Ag_2_O remains at 600 °C. Therefore, until 600 °C, as the silver content increases, the amount of Ag_2_O increases and adversely affects the electrical conductivity of the thin film.

In general, the amount of silver on the thin film plays a huge role in electrical conduction properties. When Ag is present in a small amount, it exhibits discontinuous island films due to the Volmer-Weber (island) growth mode due to the surface energy difference, which is known to have a negative effect on conductivity [49,50]. Considering this point of view, the reason why the difference between the resistivity value of ITO thin films and the one of ITO@Ag (7.5 wt.%) gets smaller and the resistivity value decreases as the heat treatment temperature rises is that the amount of Ag reduced from Ag_2_O gradually increases. The amount of reduced Ag is not enough to connect each other until 600 °C, silver still has an island shape and forms a discontinuous high resistivity thin film, which shows an upward tendency of resistivity as the introduced amount of silver increases. This can also explain why the resistivity decreases as the amount of silver increases in the heat treatment of 750 °C, which is because the amount of silver reduced at 750 °C is sufficiently large so that the silver fills in the gaps between the ITO particles and can form a continuous film.

**Figure 8:**
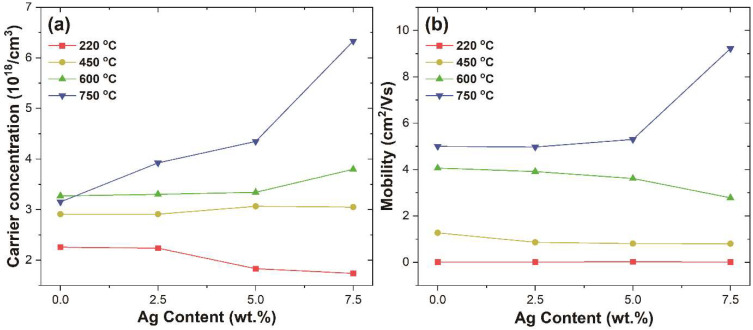
(**a**) Carrier concentration and (**b**) mobility of ITO and ITO@Ag thin films according to the silver weight percentage and thermal-treatment temperature.

**Figure 9:**
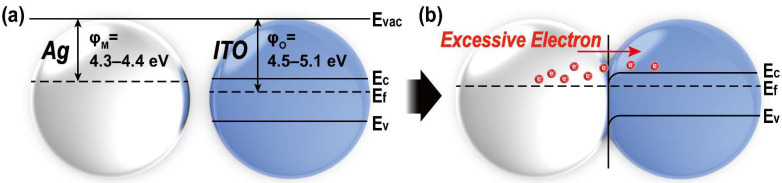
The energy band diagram for ITO and Ag (**a**) before and (**b**) after the junction formed.

It is also well known that these changes in resistivity are also related to changes in carrier concentration and carrier mobility. The measured carrier concentration (n) and mobility (μ) according to the weight percentage of silver and thermal treatment temperature are shown in Figure 8a–b, respectively. As seen in the resistivity analysis, both n and μ are also varied according to the heat-treatment temperature and weight percentage of silver. In the case of ITO, which does not contain silver, the n value tends to increase as the heat treatment temperature rises, and then it drops in 750 °C. In comparison, the μ values increase continuously as the temperature increases. As in other literature, the reason that the conductivity improvement as the heat treatment temperature of the ITO thin film increases is not due to the increment of carrier concentration but due to the increase of mobility caused by the improvement of crystallinity [51]. In contrast with other literature, the reason that the carrier concentration value transition occurs at a relatively high 600–750 °C is that a sufficient temperature is required to decompose the coupling agent acting as an electrical insulator. In the same context, since the thermal decomposition of the coupling agent and Ag_2_O did not occur at 220 °C, the *n* value tends to decrease as the silver content increases, and the μ is very small. However, as the thermal decomposition of Ag_2_O occurs at 400 °C [52,53], the n value increases along with the silver content increasing from 450 °C. This also proves that the metal incorporated with the metal oxide can be an electron source for conduction, which shows the same trend as the TCO/metal/TCO structure [54]. In the case of the μ, it decreases as the weight percentage of silver increases up to 600 °C, which, as mentioned above, is due to the formation of a p-n junction between Ag_2_O and ITO particles. As the heat treatment temperature reached 750 °C, sufficient metallic Ag was formed, so both μ and *n* increase. Moreover, the work function value of Ag ($\varphi_{M}=4.3-4.4\;\mathrm{eV}$) is smaller than the work function value of ITO ($\varphi_{O}=4.5-5.1\;\mathrm{eV}$) (Figure 9), which causes an ohmic contact, where the electron band of ITO bends down toward silver, and thus electron carriers in silver can easily move toward ITO [55,56].

**Figure 10:**
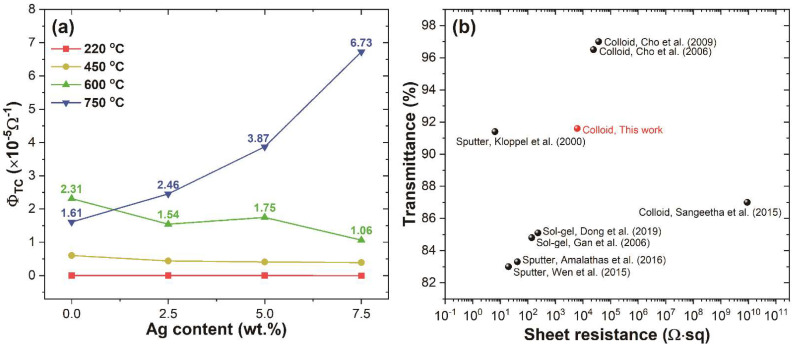
(**a**) The figure of merit (FOM) of ITO and ITO@Ag thin films according to the weight percentage of silver and thermal-treatment temperature, and (**b**) graphical representation of optical transmittance and sheet resistance for sputter, data from Refs. [54,61,62], sol-gel, data from Refs. [63,64], and colloid processed, data from Refs. [58,59,60] ITO thin films.

To quantitatively evaluate the performance of transparent conductive films with different resistivity and transparency, the figure of merit (FOM, $\Phi_{TC}$) was calculated using the equation proposed by Haacke [57].

(4) $$\Phi_{TC}=\frac{T^{10}}{R_{Sh}}$$

where $R_{Sh}$ is the sheet resistance and *T* represents the transmittance of ITO, ITO@Ag thin films at the 550 nm wavelength. In Figure 10a, the maximum FOM value was obtained as $6.73\times 10^{-5}{\;\Omega }^{-1}$ from ITO@Ag (7.5 wt.%) thin films heat-treated at 750 °C. Furthermore, as the temperature of thermal treatment increases, the FOM value tends to increase, resulting from increased carrier concentration and mobility.

Furthermore, the resistivity of our colloidally prepared ITO@Ag (7.5 wt.%) thin-film is almost 0.15, 0.3, and even 7×10^−7^ times lower than the previously reported values for colloidal ink, as shown in Figure 10b [58,59,60]. The improved electrical properties of ITO@Ag composite thin films can be summarized by the following factors: (i) As the thermal treatment temperature increases, silver oxides are reduced into silver, filling the gap between the ITO nanoparticles to work as a carrier bridge. (ii) The electrical junction built-in the interfaces between silver and ITO have the ohmic junction, making fluent transports of charge charrier from silver to ITO. (iii) Silver metal could also work as a source for supplying sufficient charge carrier to the thin films, which results in increased carrier concentration.

## 4. Conclusions

In this study, simple, highly reproducible, colloidally dispersed ITO@Ag inks were prepared through a simple high-speed ball milling process and fabricated using a spin coating method. Due to structural and electronic effects, the ITO@Ag thin films showed better electrical conduction properties than previously reported colloidal thin films. Increasing the weight percentage of silver and thermal treatment temperature lowered the resistivity of thin film to $1.07\times 10^{-1}\;\Omega \cdot \mathrm{cm}$ and high transmittance to 91.6% at 550 nm. Thus, colloidal ITO@Ag ink can be a promising method for tuning the electrical properties of transparent conductive electrodes for OLEDs (organic light-emitting diodes), touchscreens, and photovoltaics.

## Data Availability

The data presented in this study are available on request from the corresponding author.
